# Phenotypic and Metabolic Variations in High-Risk Clones of Multidrug-Resistant *Pseudomonas aeruginosa*

**DOI:** 10.3390/microorganisms14030699

**Published:** 2026-03-20

**Authors:** Sonia J. Gutierrez, Juan David Escobar Prieto, Deninson Alejandro Vargas, Richard Burchmore, Karl Burguess, Adriana Correa

**Affiliations:** 1 Biology Department, Faculty of Natural and Exact Sciences, Universidad del Valle, Cali 760031, Colombia; sonia.gutierrez@correounivalle.edu.co; 2 Biological and Environmental Science and Engineering Division, King Abdullah University of Science and Technology (KAUST), Thuwal 23955-6900, Saudi Arabia; juan.escobarprieto@kaust.edu.sa; 3 Centro de Internacional de Entrenamiento e Investigaciones Médicas (CIDEIM), Cali 760001, Colombia; 4 Universidad Icesi, Cali 760031, Colombia; 5 School of Infection & Immunity, College of Medical, Veterinary & Life Sciences, University of Glasgow, Glasgow G12 8QQ, UK; 6 Institute of Quantitative Biology, Biochemistry and Biotechnology, School of Biological Sciences, University of Edinburgh, Edinburgh EH8 9YL, UK; 7 Faculty of Basic Sciences, Universidad Santiago de Cali, Cali 760011, Colombia

**Keywords:** HRC, multidrug-resistant *Pseudomonas aeruginosa*, metabolism, virulence, intracellular survival

## Abstract

The global spread of high-risk clo1nes (HRCs) of multidrug-resistant (MDR) *Pseudomonas aeruginosa* has hindered infection control and treatment strategies worldwide. In Colombia, globally relevant HRCs such as ST235 and ST111 have been widely reported. In this study, we evaluated phenotypic and metabolic variations associated with intracellular survival and dissemination in *P. aeruginosa*. A total of 100 clinical isolates were collected from 22 hospitals in Colombia. The isolates had been previously characterized and classified as MDR or susceptible strains (SSs), and their sequence types (STs) had been earlier determined. Based on this prior characterization, isolates were grouped in this study as multidrug-resistant high-risk clones (HRC, *n* = 50; corresponding to sequence types ST235 and ST111), multidrug-resistant non-high-risk clones (NHRCs, *n* = 27; non-ST235/ST111), and susceptible strains (SS, *n* = 23; also, non-ST235/ST111). Phenotypic traits, including motility, spontaneous mutation frequency, biofilm formation, and pigment production, were evaluated. In addition, a subset of 30 isolates was assessed for intracellular survival in vitro and metabolomic profiling using liquid chromatography-mass spectrometry. HRC isolates exhibited significantly reduced motility compared with NHRC and SS isolates (swarming: HRC vs. NHRC, *p* = 0.0032; HRC vs. SS, *p* = 0.010; swimming: HRC vs. NHRC and SS, *p* < 0.0001; twitching: HRC vs. SS, *p* = 0.0004), as well as lower pigment production (pyocyanin: HRC vs. NHRC and SS, *p* < 0.0001; pyoverdine: HRC vs. NHRC, *p* < 0.0001). Metabolomic analysis revealed increased concentrations of metabolites associated with iron acquisition and siderophore-related pathways in HRC isolates. Overall, these findings suggest that *P. aeruginosa* HRCs display distinct phenotypic and metabolic patterns that may contribute to persistence and dissemination in clinical settings, contributing to their epidemiological success.

## 1. Introduction

*Pseudomonas aeruginosa* is an opportunistic bacterium that causes healthcare-associated infections [1,2]. This microorganism exhibits remarkable versatility conferred by a repertoire of virulence determinants and both intrinsic and acquired resistance to antibiotics [3]. This contributes to the development of multidrug-resistant (MDR) or extensively drug-resistant bacterial clones, termed high-risk clones (HRCs), which exhibit increased persistence and virulence, resulting in high morbidity and mortality [4,5]. Among their resistance mechanisms, the production of enzymes such as β-lactamases has emerged as the most prevalent and of clinical importance [6,7]. The overall success of these clones stems from the complex interplay between pathogenicity, endemicity, and antibiotic resistance [8,9]. Understanding the phylogenetic relationships of these carbapenemase-producing MDR pathogens and their distribution in the population has facilitated the exploration of the epidemiological relationships of infections caused by these microorganisms [10]. Molecular tools such as pulsed-field gel electrophoresis (PFGE) and multilocus sequencing typing (MLST) have identified successful MDR bacterial lineages or sequence types, which can adapt to the hospital environment for extensive periods and disseminate globally across regions [11]. Interestingly, NHRC *P. aeruginosa* also shares identical resistance profiles and mechanisms with those found in HRCs, indicating the existence of other factors beyond antimicrobial resistance that confer the ability to spread and persist but that remain poorly understood [9,12].

In 2013, Mulet et al. [9] reported that while *P. aeruginosa* HRCs are significantly associated with higher levels of biofilm formation and spontaneous mutation frequencies, they have lower motility (swimming, swarming, and twitching) and pigment production (pyoverdine and pyocyanin). These biomarkers or virulence factors effectively contribute to the pathogenesis of *P. aeruginosa* that facilitate tissue invasion and damage, immune evasion, and antimicrobial resistance [3,13,14,15]. In 2020, Del Barrio-Tofiño et al. [16] identified the top 10 *P. aeruginosa* HRCs associated with MDR and poor clinical prognosis as ST235, ST111, ST233, ST244, ST357, ST308, ST175, ST277, ST654, and ST298 [16,17,18,19,20].

Clones ST235 and ST111, among others, are the most widely disseminated in Colombia; thus, further exploration is required to ascertain their transmission dynamics [21]. However, few studies have jointly evaluated the phenotypic and metabolic profiles in clinically relevant isolate collections. Therefore, this study assessed the phenotypic traits and metabolomic patterns in a multicenter Colombian collection of multidrug-resistant HRCs, NHRCs, and susceptible isolates to identify group-level differences and exploratory metabolic signatures potentially associated with dissemination and intracellular survival.

## 2. Materials and Methods

### 2.1. Bacterial Strains

One hundred clinical isolates of *P. aeruginosa* stored in the biobank of the Centro Internacional de Entrenamiento e Investigaciones Médicas (CIDEIM) and originally collected from 22 hospitals in Colombia were selected using convenience sampling. The isolates had been previously identified and classified as multidrug-resistant (MDR) or susceptible strains (SSs). Additionally, information regarding multilocus sequence typing (MLST) and the presence of carbapenemases was available from previous characterizations recorded in the biobank database (Supplementary Table S1). Based on these records, the isolates were categorized for the purposes of this study into MDR high-risk clones (HRCs), MDR non–high-risk clones (NHRCs), and susceptible strains (SS). The number of isolates in each group was as follows: HRCs (*n* = 50; ST235/ST111), NHRCs (*n* = 27; non-ST235/ST111), and SSs (*n* = 23; non-ST235/ST111).

### 2.2. Motility Evaluation

Swarming, swimming, and twitching behaviors were assessed in accordance with the methodologies outlined by Mulet et al. [9] and Filloux [22], with some modifications. Swarming motility was assessed by seeding 1 μL of the bacterial inoculum (optical density at 600 nm [OD600] = 1.5) onto a Luria–Bertani (LB) broth Difco, BBL™, Franklin Lakes, NJ, USA-based plate containing M8 swarming medium (M9 salts without NH_4_Cl, supplemented with 1 mM MgSO_4_, 0.2% glucose, 0.5% casamino acids, and 0.5% W/V agar) [9,22,23,24]. The bacteria were then incubated overnight in a humid chamber. The length of the dendritic colony projections that formed on the agar surface was measured in millimeters (mm) [25]. Swimming motility was assessed using a lower-concentration swim medium (M8 with 0.3% W/V agar). A bacterial suspension from the LB broth was inoculated by pricking the agar up to half its thickness using a sharp sterile toothpick. The extent of radial colonial growth in mm was then measured after incubation. Twitching motility was also assessed using stab inoculation. A bacterial colony picked up with a sterile toothpick was inoculated onto 1% LB agar by pricking the agar until contact with the Petri dish base was made. The diameter in mm of the halo observed at the agar interface was then measured. All motility inoculations were incubated at 37 °C for 24 h and performed in triplicate, and the mean value obtained from these replicates was used for the statistical analyses.

### 2.3. Pigment Production

Pyoverdine and pyocyanin production was quantified as previously described with some modifications [9,26]. The strains were grown in tubes containing 2% casamino acid broth supplemented with 5.4% K_2_HPO_4_ and 0.025% MgSO_4_·7H_2_O and incubated at 37 °C for 40 h. The isolates were centrifuged at 5000 rpm for 5 min to remove bacterial cells and obtain clarified supernatants, minimizing potential interference from turbidity during spectrophotometric measurements. Subsequently, 150 µL of the supernatant was dispensed into microplates. The amount of pyocyanin (blue pigment) was determined by measuring the absorbance of the supernatants at 580 nm. The amount of pyoverdine was determined by fluorescence emission at a wavelength of 505 nm with an excitation of 405 nm. Each experiment was performed in six replicates, and the mean value obtained from these replicates was used for the statistical analyses.

### 2.4. Biofilm Formation

Biofilm formation was evaluated as described by Merritt and Kadaouri [27] with some modifications. Briefly, 100 µL of the bacterial inoculum (OD600 = 0.05) was dispensed into microplates with 150 µL LB broth per well, which was then capped, and the inoculations were incubated at 37 °C for 48 h. The plates were then washed with tap water to remove planktonic cells, dried, and added with 125 µL of 0.1% crystal violet solution to each well for 10 min at room temperature. The plates were rinsed, dried again, and then added with 150 µL of 30% acetic acid at room temperature for 25 min. The inoculations were subsequently homogenized with a multichannel micropipette, and 125 µL per well was dispensed into a 96-well microplate, with the absorbance measured at 550 nm. Six replicates were performed, and the mean value was used for statistical analysis. Finally, biofilm production of the isolates was categorized using the method of Stepanovic et al. [28] as strong, moderate, weak, or non-biofilm-producing by comparing the OD of each isolate to that of the negative control, which was the uninoculated culture medium (ODc).

### 2.5. Determination of the Frequency of Spontaneous Mutations

The frequency of mutation in response to rifampicin (100 µg/mL on each agar; Sigma-Aldrich, St. Louis, MO, USA) was determined using a modified version of protocols previously described by Mulet et al. [9] and Oliver [14]. Each strain was diluted to 1:70,000, starting from a 0.5 Mc Farland inoculum. Then, 100 µL of the dilution was plated on LB agar and inoculated into LB broth Difco, BBL™, Franklin Lakes, NJ, USA at 37 °C for 24 h. Subsequently, 500 µL was taken from the broth and agar and seeded onto LB–rifampicin agar (LB-Rif) for 48 h at 37 °C. Mutation frequencies were estimated by dividing the average number of colonies in LB-Rif (mutants) by the average number of colonies in LB agar.

### 2.6. Evaluation of Intracellular Survival

A subset of 30 isolates was selected as representative of the three groups, including 9 NHRCs, 11 HRCs, and 10 SSs, for the evaluation of intracellular survival and persistence. Intracellular survival was assessed using the human monocytic cell line THP-1 (ATCC TIB-202; American Type Culture Collection, Manassas, VA, USA). Cells were cultured in Roswell Park Memorial Institute (RPMI) 1640 medium (Gibco, Thermo Fisher Scientific, Waltham, MA, USA) supplemented with 10% fetal bovine serum (FBS; Gibco, Thermo Fisher Scientific, Waltham, MA, USA) and differentiated into macrophage-like cells using phorbol 12-myristate 13-acetate (PMA; Sigma-Aldrich, St. Louis, MO, USA) at 100 ng/mL for 24 h, as previously described [29,30]. Bacterial isolates were opsonized for 1 h in RPMI 1640 medium containing 10% human AB serum Sigma-Aldrich, St. Louis, MO, USA (without FBS). Opsonized bacteria were then added to differentiated THP-1 cells at a 1:1 bacteria-to-cell ratio and incubated for 2 h to allow phagocytosis. To eliminate extracellular and non-adherent bacteria, infected cells were treated with gentamicin (100 μg/mL; Sigma-Aldrich, St. Louis, MO, USA) and polymyxin B (8 μg/mL; Sigma-Aldrich, St. Louis, MO, USA). To test 24-h intracellular survival, infected cells were maintained in the presence of these antibiotics for 24 h. After incubation, cells were washed twice with phosphate-buffered saline (PBS; Gibco, Thermo Fisher Scientific, Waltham, MA, USA), scraped, and sonicated for 10 s to release intracellular bacteria. The resulting lysates were diluted 1:10 and plated on Mueller–Hinton agar (MHA; Becton Dickinson, Franklin Lakes, NJ, USA) to determine colony-forming units (CFUs).

### 2.7. Preparation and Culture of P. aeruginosa Clones, Metabolite Extraction, and LC-MS/MS Analysis

#### 2.7.1. Bacterial Cell Culture Preparation

Thirty clinical *P. aeruginosa* isolates were randomly selected from the total population previously characterized phenotypically and genotypically (*n* = 100) and categorized into 10 HRCs, 10 NHRCs, and 10 SS isolates (Supplementary Table S1). The isolates were then prepared using a previously described protocol [31] that was adapted to *P. aeruginosa*. Briefly, an inoculum (OD600 = 0.1) was prepared from pure cultures in 0.85% NaCl. Then, 1 mL of the inoculum was added to 10 mL LB broth and incubated at 37 °C (18–20 h). After incubation, 3 mL was centrifuged at 4 °C at 6500 rpm for 10 min. The resultant pellet was resuspended in 5 mL of cold 0.85% NaCl, centrifuged twice at 6500 rpm for 5 min, and resuspended in 1 mL cold 0.85% NaCl. Finally, the suspension was adjusted to OD_600_ = 1.0 with saline, 1 mL of which was centrifuged at 13,000 rpm for 5 min.

#### 2.7.2. Preparation of Metabolites by Shaking with Beads

The pellet was resuspended in 200 µL at a 1:3:1 ratio of chloroform:methanol:water and vigorously shaken. Glass beads (1 g) were added to the suspension and adjusted to 1 mL with the chloroform:methanol:water mixture. The suspension was vortexed continuously at 3000 rpm at 4 °C for 10 min. Finally, it was centrifuged at 13,000 rpm at 4 °C for 10 min. The supernatant was collected and stored at −80 °C until analysis. The samples were analyzed by hydrophilic interaction liquid chromatography-mass spectrometry (HILIC-LC-MS). The results were analyzed using Polyomics integrated Metabolomics Pipeline (PiMP) available at http://polyomics.mvls.gla.ac.uk, accessed on 26 January 2026 (Glasgow Polyomics, Glasgow, UK) and MetaboAnalyst 4.0 (Glasgow Polyomics, Glasgow, UK).

#### 2.7.3. LC-MS/MS Data Acquisition

The samples were analyzed by LC-MS/MS for hydrophilic interaction (UltiMate 3000 RSLC system, Thermo Fisher Scientific, Germering, Germany) using a ZIC-pHILIC column (Merck SeQuan, Darmstadt, Germany, 150 × 4.6 mm) operated at 300 µL/min with ion detection by Orbitrap Exactive (Thermo Fisher Scientific, Bremen, Germany). The mass spectrometer was operated at 50,000 resolving power in the positive/negative exchange mode. The buffer solutions consisted of (A) 20 mM ammonium carbonate (Sigma, St. Louis, MO, USA) dissolved in H_2_O and (B) Merck SeQuant acetonitrile (Rathburn Chemicals, Peeblesshire, UK). The LC gradient was programmed as follows: from 20% A:80% B to 80% A:20% B for 15 min, followed by a wash at 95% A:5% B for 3 min, and equilibrium at 20% A:80% B for 5 min. Raw data were processed using XCMS v.1.0.0. [32], MzMatch v.2.0-6 [33] and internally developed R codes for filtering, post-processing, and identification. The data were compiled and visualized using PiMP v.3.0 [34]. Metabolites were classified based on the Metabolomics Standards Initiative guidelines [35]. Compounds were considered “identified” if they met the criteria of <3 ppm mass precision and <5% retention time error.

#### 2.7.4. Data Quality Control

Equal aliquots of each sample were pooled to generate the control master sample (QC). All samples were analyzed in 1 day and in a single, randomized batch. Stock solutions of 116 authentic standards were prepared in 50% ethanol/water or Milli-Q water, depending on the solubility. A working solution with all authentic standards was prepared and injected at the beginning and end of each run [36]. The QC was injected before starting the LC-MS/MS run and every five samples thereafter to ensure analytical stability. The quality of the chromatograms and reproducibility of the signal was tested by analyzing the QC samples. Once the data coupling had been performed, a database of 2179 detectable features corresponding to 11,642 metabolites was obtained, including isomers and duplicate entries (Supplementary Table S2). Manual curation of the data was required to confirm the annotated and identified metabolites. All detected peaks that did not allow the annotation of metabolites were discarded from the subsequent analyses, retaining only those peaks with annotated or identified metabolites.

### 2.8. Statistical Analysis

To analyze the phenotypic tests, the normality of the variables was assessed using the Shapiro–Wilk test, and because the data did not follow a normal distribution, non-parametric tests were applied. Differences among the three clonal groups (HRC, NHRC and SS) were evaluated using the Mann–Whitney U test. All statistical analyses were conducted using STATA^®^ v. 12 at a significance level of *p* < 0.05, and graphs were constructed using the GraphPad Prism 6 tool (GraphPad Software Inc., San Diego, CA, USA).

For the metabolomic component, once the cleaned database was obtained, metabolites were analyzed using principal component analysis (PCA) and analysis of variance (ANOVA) in the web-based software MetaboAnalyst 4.0 [37]. GraphPad Prism 6 was used to complement statistical analysis. Peak intensities were normalized using the probabilistic quotient [38], generalized log-transformed, and scaled using the Pareto method [39]. Multivariate analyses (ANOVA and PCA) were performed to identify metabolites associated with the phenotypes of interest. Metabolomic network analysis was performed using the Kyoto Encyclopedia of Genes and Genomes, MetaboAnalyst 4.0, and MetaCyc v.21.5.

## 3. Results

### 3.1. High-Risk Clones Exhibited Reduced Motility

*P. aeruginosa* exhibits swimming, swarming, and twitching motilities. The flagellum facilitates the first two, and the type IV pili facilitates twitching motility [15,40,41]. Furthermore, the swimming motility of the bacterium is essential to induce neutrophil extracellular traps (NETs). NETs are web-like chromatin structures that function in the trapping and elimination of microorganisms. Dysregulation of this process has been implicated in the pathogenesis of diseases, such as cystic fibrosis, which is characterized by polymorphonuclear leukocyte-dominated inflammation [42,43].

**Figure 1:**
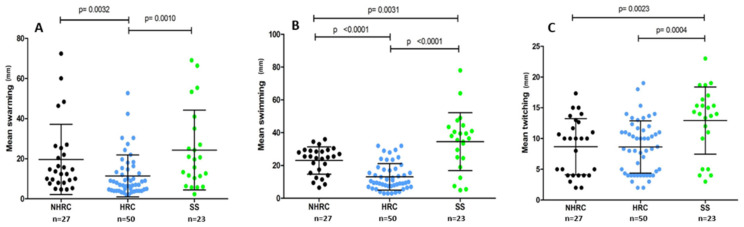
Motility screening of the different isolates according to (**A**) swarming, (**B**) swimming, and (**C**) twitching. Mean diameter is expressed in millimeters of NHRC (black), HRC (blue), and SS (green) clones (*p*-value, Mann–Whitney U test).

The evaluation of motility phenotypes revealed significant differences among HRC vs. NHRC isolates (*p* = 0.0032) and HRC vs. SS (*p* = 0.010), specifically for swarming (Figure 1A). This same phenomenon was observed in swimming (Figure 1B), with significant differences between HRC vs. NHRC (*p* < 0.0001) or HRC vs. SS (*p* < 0.0001). Pairwise comparisons between groups were performed using the Mann–Whitney U test.

Twitching motility, which is linked to type IV pili function (Figure 1C), is generally impacted in resistance strains regardless of clone type. Significant differences were noted between sensitive isolates and HRC or NHRC, respectively (SS vs. HRC *p* = 0.0004 and SS vs. NHRC *p* = 0.0023, Mann–Whitney U test).

### 3.2. HRCs Showed Lower Pyocyanin and Pyoverdine Production

**Figure 2:**
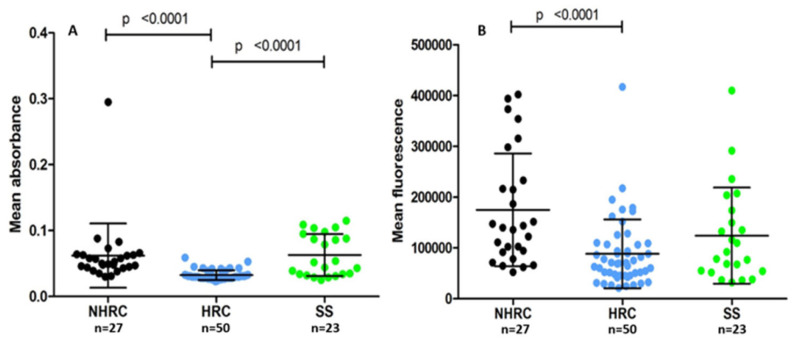
Pigment production by the different *Pseudomonas aeruginosa* isolates. (**A**) Mean absorbance per clone for pyocyanin production. (**B**) Mean fluorescence per clone for pyoverdine production. NHRC (black), HRC (blue), and SS (green) (*p*-value, Mann–Whitney U test).

Among the *P. aeruginosa* virulence factors involved in colonization, infection development, and nutrient acquisition are the pigments pyocyanin and pyoverdine [44,45,46]. HRC isolates exhibited lower pyocyanin production than NHRC and SS (HRC vs. NHRC *p* < 0.0001 and HRC vs. SS *p* < 0.0001, Mann–Whitney U test; Figure 2A). This difference was not observed between the NHRC and SS clones. As with pyoverdine, HRC isolates showed a lower production of pyoverdine than NHRC (*p* < 0.0001, Mann–Whitney U test; Figure 2B). These results indicate that HRC isolates decreased the expression of these virulence factors.

### 3.3. High-Risk Clones Show Similar Behavior to Sensitive Isolates in Biofilm Production

**Figure 3:**
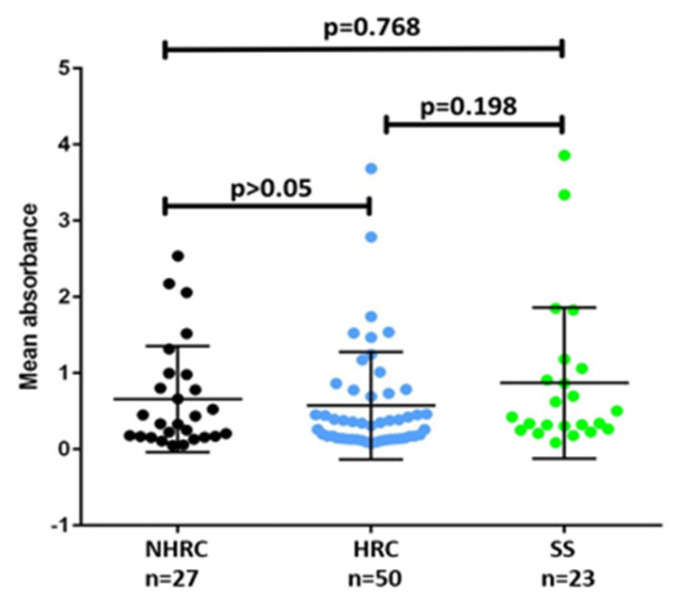
Biofilm formation by the different *Pseudomonas aeruginosa* isolates. Average absorbances exposed by NHRC (black), HRC (blue), and SS (green) clones. (*p*-value, Mann–Whitney U test).

One of the key characteristics contributing to the pathogenicity of *P. aeruginosa* is its biofilm formation ability, a strategy that facilitates colonization in diverse environments [43,44]. This capability is linked to increased tolerance and resistance to antimicrobials and disinfectants, evasion of immune system responses, persistence of chronic infections, and challenges to treatment [47,48,49,50,51,52]. Using the method by Stepanovic et al. [28], most isolates were categorized as strongly adherent (54%), with HRCs showing the highest proportion of this phenotype. Only 1% of the isolates were identified as non-biofilm-forming. However, no significant differences were found among the HRCs, NHRCs, and SSs (*p* > 0.05 Mann–Whitney U test; Figure 3).

### 3.4. Spontaneous Mutations in P. aeruginosa Occurred Independently of Antimicrobial Resistance

**Figure 4:**
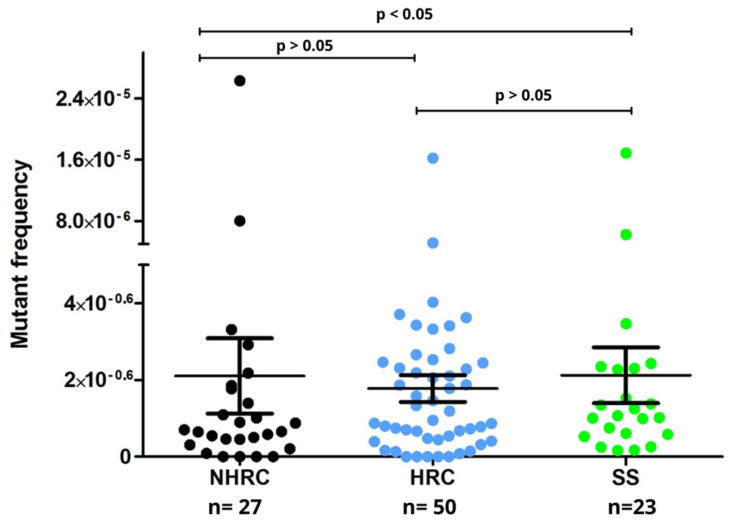
Spontaneous mutation frequency by the different *Pseudomonas aeruginosa* isolates. Mutation frequency obtained by NHRC (black), HRC (blue), and SS (green) clones (*p*-value, Mann–Whitney U test).

Mutations are bacterial adaptive strategies in response to stressful environments, such as antibiotic exposure or extreme starvation [53]. Environmental changes can induce genetic variations, causing mutations that result in novel phenotypes [53,54,55]. In the present study, the frequency of spontaneous mutations was remarkably similar across the different groups (HRC, NHRC, and SS), suggesting that this strategy may be adopted by strains linked to acute infections and occurs independently of their antibiotic resistance profiles. However, no significant differences in mutation frequency were observed among the groups (*p* > 0.05, Mann–Whitney U test; Figure 4).

### 3.5. Intracellular Survival Was Lower in SS Isolates with Respect to MDR (HRC and NHRC)

**Figure 5:**
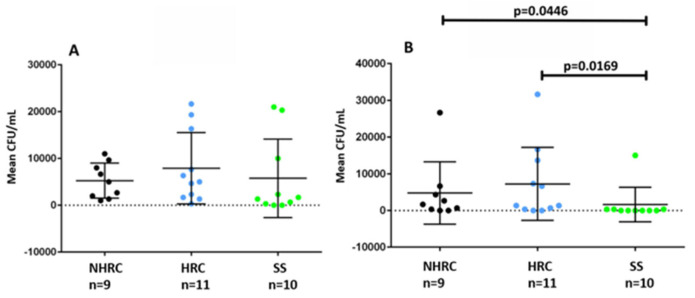
Intracellular survival assay in human monocytic THP-1 cells by the different *Pseudomonas aeruginosa* isolates. (**A**) Assessment of infection after 2 h of incubation with the cells. (**B**) Assessment of intracellular survival after 24 h. Mean CFU/mL for NHRC (black), HRC (blue), and SS (green) clones. (*p*-value, Mann–Whitney U test).

Although *P. aeruginosa* is an extracellular pathogen, clinical isolates are able to internalize and survive in host cells and animal models, contributing to pathogenesis and antimicrobial therapy evasion [29,56,57,58]. This study evaluated the ability of *P. aeruginosa* to infect and persist in THP-1 cells in vitro. No significant differences among the clonal groups were observed 2 h after infection (Figure 5A). However, when evaluating intracellular survival at 24 h (Figure 5B), significant differences were found between NHRC vs. SS (*p* = 0.0446) and between HRC vs. SS (*p* = 0.0169, Mann–Whitney U test).

### 3.6. Metabolomic Analysis in High-Risk Clones of P. aeruginosa

**Figure 6:**
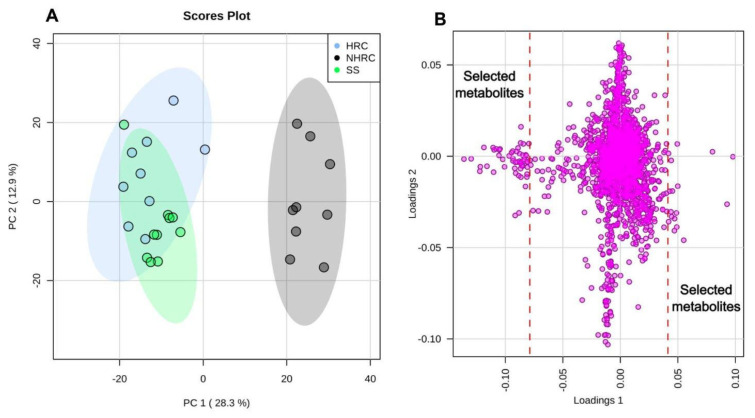
Metabolomic analysis of the *Pseudomonas aeruginosa* strains. (**A**) Principal component analysis (PCA), each point represents a strain. Blue (HRC); black (NHRC); green (SS): antibiotic-sensitive *P. aeruginosa* strains. PC1: principal component 1 represents the percentage of variance that explains the separation of the strains on the *x*-axis. PC2: principal component 2 represents the percentage of variance explaining the separation of the strains on the *y*-axis. The ovals represent the 95% confidence intervals. (**B**) Dot plot showing the weight (loadings) of each metabolite in explaining the separation of the groups on the *x*- and *y*-axes. The dashed red lines indicate the cutoff points established (−0.08 ≤ loadings1 ≤ 0.04) to select the metabolites that best explain the separation of the groups on the *x*-axis. Data were normalized using the SS group as a control, log-transformed, and scaled using the Pareto method. NHRC, non-high-risk clones; HRC, high-risk clones; SS, susceptible strains.

The metabolomic data obtained from the strains (HRC, *n* = 10; NHRC, *n* = 10; and SS, *n* = 10) indicated that two strains (one HRC and one NHRC) should be excluded from further analysis, as they were identified as outliers by PCA using a 95% confidence interval. PCA also showed a distinct separation between the HRC and NHRC groups, with PC1 explaining 28.3% of the variance (Figure 6A). The susceptible strains, included as controls, showed overlapping patterns with the HRC strains (Figure 6A). Overall, these results suggest comparable metabolomic profiles between the HRCs and SSs, with a notable divergence from the NHRC strains. This pattern may help explain the enhanced dissemination capacity of HRCs.

Selection of the metabolites contributing most strongly to group separation was performed using the loadings plot, with cutoff points defined as −0.08 ≤ loadings1 ≤ 0.04 (Figure 6B). Metabolites with an ANOVA *p*-value ≤ 0.05 were retained, yielding 73 unique peaks. Of these, 40 could not be assigned to a specific metabolite and were therefore excluded from further analysis. The remaining 33 unique peaks (metabolites) were used for downstream pathway analysis to identify differentially modulated metabolic pathways.

**Figure 7:**
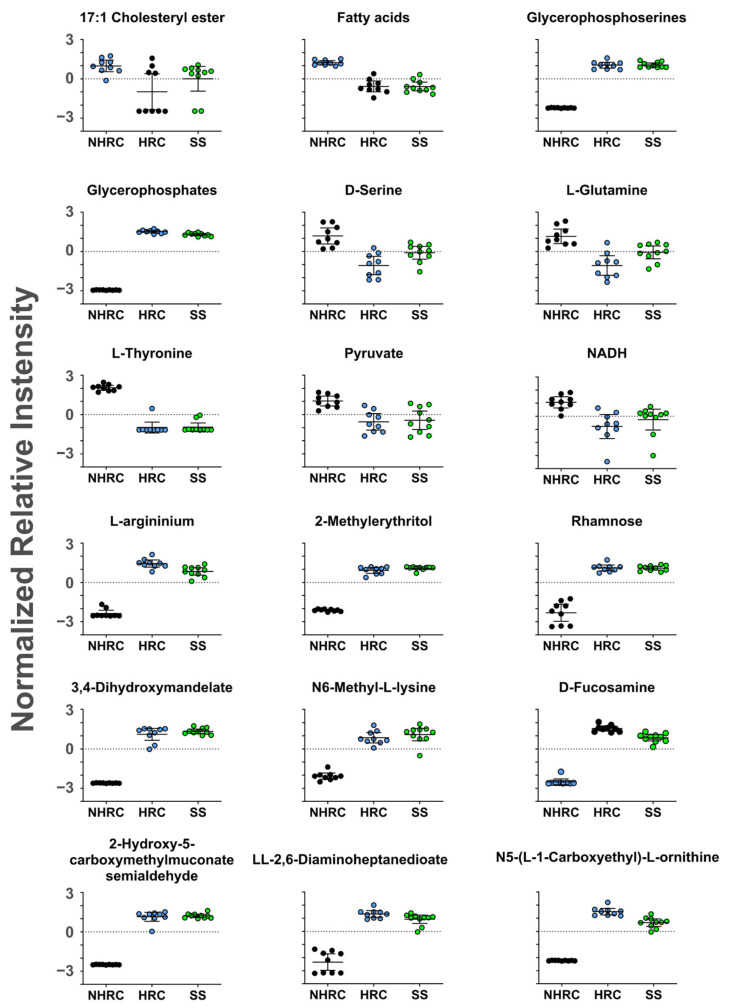
Analysis of selected metabolites in the different strains. NHRC (black); HRC (blue); SS (green). All metabolites were significant (*p* < 0.05) in the NHRC vs. HRC comparisons. NHRC, non-high-risk clones; HRC, high-risk clones; SS, susceptible strains.

Analysis of enriched metabolic pathways did not identify any pathway with significant differences between groups. However, a clear trend was observed in NHRCs toward glucogenic amino acid metabolism (serine, threonine, and glutamine) and energy-related intermediates such as pyruvate and NADH (Figure 7). In addition, NHRCs showed a higher accumulation of fatty acids, which are key precursors for phospholipid synthesis. Notably, this fatty acid accumulation was not accompanied by increased phospholipid levels (glycerophosphates and glycerophosphoserines), which would be expected if these substrates were being incorporated into phospholipids or utilized through oxidative catabolic processes (β-oxidation). In contrast, HRCs exhibited higher phospholipid concentrations, as well as increased levels of 2-methylerythriol, an intermediate in isoprenoids biosynthesis [57], which are essential compounds for bacterial growth (Figure 7). *P. aeruginosa* HRCs also presented higher levels of metabolites related to virulence and pathogenicity, such as rhamnose (Figure 7).

## 4. Discussion

The present study revealed that *P. aeruginosa* HRCs displayed impaired swimming, swarming, and twitching motilities compared with the NHRCs and SS isolates; this reduction may represent an adaptive response that prioritizes persistence and stability over dissemination. Although reduced motility has been linked to biofilm-related persistence in prior studies, our data did not show significant differences between groups in biofilm biomass and therefore, these relationships should be interpreted cautiously in the context of our collection [9,40,59,60,61,62,63,64,65,66,67,68,69,70,71,72].

Pigment production analysis revealed lower pyoverdine and pyocyanin production in the HRCs than in the NHRCs and SSs. These pigments have been reported to contribute to iron acquisition, oxidative stress modulation, and virulence regulation while interfering with various host cellular functions [44,45,46,73,74,75,76]. Thus, their downregulation in HRCs may indicate an energy-saving mechanism that favors long-term survival [45,77]. Similar observations have been reported in other *P. aeruginosa* MDR isolates with attenuated pigment synthesis, signifying potential metabolic reprogramming toward persistence rather than acute virulence [9,73,74]. However, pigment levels in the present study were estimated by direct spectrophotometric and fluorescence measurements of clarified culture supernatants, which provide a comparative approximation but may be influenced by other secreted metabolites; therefore, the results should be interpreted primarily as relative differences among isolates rather than absolute pigment concentrations. Consequently, these findings should be interpreted as associative observations rather than direct evidence of altered oxidative stress responses.

The frequency of spontaneous mutations did not differ markedly among phenotypes, aligning with the results by Mulet et al. [9] and Oliver et al. [14]. These findings may indicate that HRC phenotypes do not rely on elevated mutation rates for adaptation. In this context, previous studies have suggested that *P. aeruginosa* populations may maintain genomic stability while acquiring adaptive traits through mechanisms such as horizontal gene transfer [9,14,53,54,55]; however, because genomic exchange processes were not evaluated in the present study, this interpretation should be considered a hypothesis and warrants further investigation.

Due to its versatility, *P. aeruginosa* can invade and survive within eukaryotic cells, which complicates eradication by antimicrobial therapy [29,56,58]. In our experiments, MDR isolates, particularly HRCs, tended to display higher intracellular survival in macrophage-like cells compared with other groups, suggesting an enhanced capacity to withstand host immune defenses; nevertheless, these findings should be interpreted with caution. The experimental design relied on extracellular antibiotic suppression to prevent the growth of non-internalized bacteria; a strategy frequently used in macrophage infection models. However, prolonged antibiotic exposure and potential effects on host cells may influence the interpretation of long-term intracellular survival measurements. Consequently, the assay provides a comparative indication of intracellular survival among the evaluated isolates rather than a direct demonstration of true bacterial persistence.

Metabolomic profiling revealed that HRCs and SS isolates have similar elevated intermediates associated with pyoverdine-related metabolism and cell wall integrity [78,79]. Consistent with this, pyoverdine production was comparable between HRCs and SS isolates in our phenotypic assays, whereas NHRC isolates showed higher levels. The abundance of rhamnose, a key component of lipopolysaccharides and rhamnolipids, may indicate differences in surface-associated functions relevant to host interaction and persistence [80]; however, because biofilm biomass did not differ between groups in our assays, this interpretation should be considered hypothesis-generating rather than definitive.

Taken together, these findings suggest that *P. aeruginosa* HRCs display coordinated phenotypic and metabolic features that may support persistence and survival in clinical environments while exhibiting reduced motility and lower pyocyanin production relative to NHRCs. This combination may contribute to the successful establishment and dissemination of HRC lineages across diverse clinical settings.

## 5. Conclusions

*P. aeruginosa* is an opportunistic pathogen with globally distributed high-risk clonal lineages characterized by multidrug resistance and diverse adaptive mechanisms. However, variations in the expression of virulence-associated traits and other adaptive phenotypes may contribute to the selection, persistence, and dissemination of these lineages in clinical environments. The present study demonstrates that HRCs display coordinated phenotypic and metabolic features that may support persistence and survival in clinical settings. In contrast, no marked differences in biofilm biomass were observed among the evaluated clonal groups, suggesting that biofilm formation was not strongly associated with resistance profile or clonality within the collection of isolates evaluated.

Higher intracellular survival observed in HRCs in macrophage-like cells at 24 h coincided with an increased level of metabolites with diverse cellular functions. In addition, spontaneous mutation frequencies and intracellular survival capacity did not appear to be associated with antimicrobial susceptibility profiles in the isolates analyzed.

Interestingly, several characteristics observed in the HRCs isolated from acute clinical infections showed similarities to those reported in chronic infections. These similarities suggest common potential adaptive strategies that enable these strains to evade the host immune response and perpetuate their persistence and dissemination. However, the findings of the present study may also reflect an early adaptation process whose clinical implications warrant further investigation.

## Data Availability

The original contributions presented in this study are included in the article/Supplementary Materials. Further inquiries can be directed to the corresponding author.

## Supplementary Materials

The following supporting information can be downloaded at: https://www.mdpi.com/article/10.3390/microorganisms14030699/s1, Supplementary Table S1: Molecular and epidemiological characteristics of the *P. aeruginosa* isolates; Supplementary Table S2: Database of metabolites obtained by metabolomics.

## Abbreviations

MLST	Multilocus sequencing typing
PFGE	Pulsed-field gel electrophoresis
HRCs	High-risk clones
MDR	Multidrug-resistant
